# Dapsone-Induced Methemoglobinemia in a Woman With Multiple Myeloma Without Glucose-6-Phosphate Dehydrogenase (G6PD) Deficiency

**DOI:** 10.7759/cureus.63249

**Published:** 2024-06-26

**Authors:** Sakshi Bai, Abraham Kisule, Bipneet Singh, Kavita Luthra, Danesh Kumar

**Affiliations:** 1 Internal Medicine, Henry Ford Jackson Hospital, Jackson, USA; 2 Nephrology, Henry Ford Jackson Hospital, Jackson, USA

**Keywords:** hypoxemia, methylene blue, saturation gap, methemoglobinemia, multiple myeloma

## Abstract

Methemoglobinemia is a condition characterized by the presence of abnormal hemoglobin, known as methemoglobin, in the blood, which impairs the ability of red blood cells to carry oxygen effectively. Symptoms include cyanosis, shortness of breath, fatigue, and in severe cases, organ damage or death. We presented a case of a 49-year-old female with multiple myeloma who developed drug-induced methemoglobinemia while on dapsone prophylaxis for Pneumocystis carinii pneumonia (PCP). Despite normal glucose-6-phosphate dehydrogenase (G6PD) levels, the patient exhibited cyanosis and shortness of breath. The case underscores the importance of considering methemoglobinemia in patients with unexplained hypoxemia, especially when associated with medication use. Diagnosis relies on clinical assessment, arterial or venous blood gas analysis with co-oximetry, and a thorough medication history. Methemoglobinemia poses a diagnostic challenge due to its varied presentations and requires a high index of suspicion, particularly in patients with multiple myeloma receiving potentially causative medications such as dapsone. Thorough evaluation, interdisciplinary collaboration, and prompt treatment are essential for favorable outcomes in these complex cases.

## Introduction

Methemoglobinemia is a condition characterized by the presence of an abnormal form of hemoglobin called methemoglobin in the blood. Hemoglobin is the protein in red blood cells responsible for carrying oxygen throughout the body. Methemoglobin forms when the iron in hemoglobin is oxidized from its normal ferrous (Fe2+) state to the ferric (Fe3+) state, making it unable to bind and transport oxygen effectively [1]. As a result, tissues may not receive an adequate supply of oxygen, leading to symptoms such as cyanosis (bluish discoloration of the skin), shortness of breath, fatigue, headache, and, in severe cases, organ damage or even death. We present a case of drug-induced methemoglobinemia in a patient on a prophylaxis dose of dapsone while undergoing therapy for multiple myeloma despite having normal glucose-6-phosphate dehydrogenase (G6PD) levels, of which only a few cases have been reported so far.

## Case presentation

**Figure 1:**
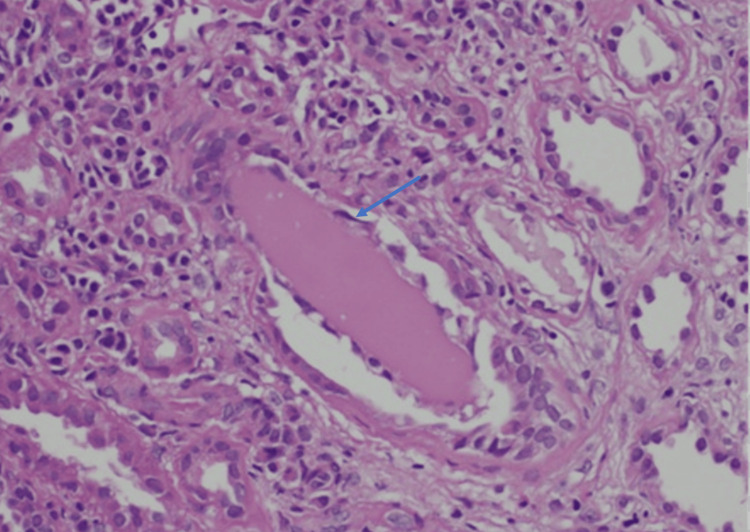
Arrow points cast nephropathy, renal consequences of monoclonal gammopathy

**Figure 2:**
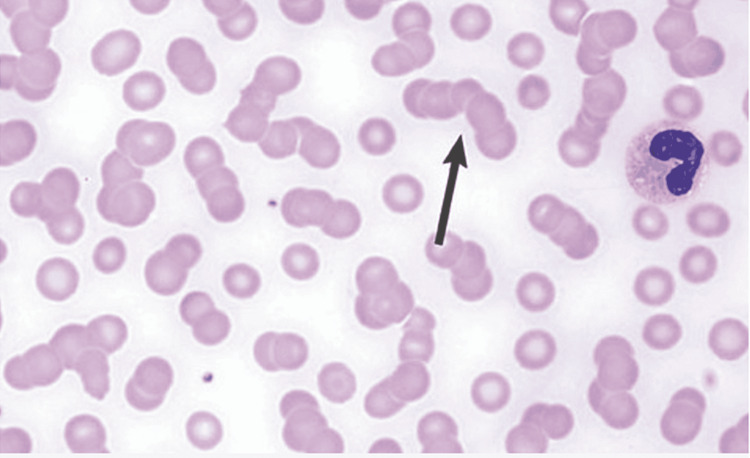
Hematological abnormalities in multiple myeloma: Rouleaux formation

A 49-year-old Caucasian female was incidentally found to have acute renal failure, with a creatinine level of 4.8 mg/dL (baseline Cr: 0.8-1.01 mg/dL), during a routine visit to her primary care physician. Subsequently, she was admitted to the hospital, where a routine workup revealed the absence of albuminuria but the presence of proteinuria. Her random urine protein ratio was 5 g, prompting suspicion of non-albumin protein excretion. Further investigations disclosed elevated kappa chains at 6,989 mg/L. A renal biopsy confirmed clear evidence of light chain cast nephropathy (Figure 1), consistent with a diagnosis of multiple myeloma. An oncologist was consulted. A further bone marrow biopsy confirmed the diagnosis of multiple myeloma, showing rouleaux formation (Figure 2), and the patient was initiated on cyclophosphamide, bortezomib, dexamethasone (CYBOR-D), accompanied by dapsone for Pneumocystis carinii pneumonia (PCP) prophylaxis, chosen due to her allergy to trimethoprim-sulfamethoxazole (TMP-SMX). G6PD levels were checked prior to initiation and were normal (G6PD levels were checked as TMP-SMX can cause hemolytic anemia in patients with G6PD deficiency). The patient was discharged with a scheduled follow-up appointment with the oncology team.

Three weeks later, she was readmitted due to complaints of shortness of breath and cyanosis of her lips and fingers. The pulse oximeter at home showed oxygen saturation (SaO_2_) at 81%. She remained hypoxic despite receiving 5-6 L of oxygen, as per pulse oximeter checks. Workup for hypoxemia, including arterial blood gas (ABGs), CT pulmonary embolism (CTPE), and chest X-ray, was unremarkable. ABGs revealed a pH of 7.48, CO_2_ of 32.9 mmHg, HCO_3_ of 24.2 mmol/L, O_2_ saturation of 97.4%, and PO_2_ of 123 mmHg. The ABG showed normal oxygen saturation; however, the pulse oximeter indicated hypoxia with SaO_2_ at 89%, indicating a saturation gap. Given the patient's history of dapsone use and the saturation gap, methemoglobinemia was suspected, and a methemoglobin level was checked, which was 16.9%. The diagnosis of methemoglobinemia was confirmed, and treatment with methylene blue and vitamin C was initiated. Treatment with methylene blue resulted in significant improvement, and the patient was discharged in satisfactory condition.

## Discussion

Methemoglobinemia can arise from congenital or acquired factors. Congenital cases stem from either an autosomal recessive mutation in the CYB5R3 gene or autosomal dominant variations in genes encoding a specific type of globin, known as hemoglobin M (HbM) disease. Hereditary methemoglobinemia linked to CYB5R3 mutations causes nicotinamide adeninedinucleotide (NADH)-cytochrome-reductase deficiency, while autosomal dominant disorders involving various globin-encoding genes lead to HbM formation, characterized by structural abnormalities, causing iron auto-oxidation and methemoglobinemia. Although cyanosis is common in these patients, they typically remain asymptomatic. Acquired methemoglobinemia results from exposure to substances, including drugs or toxins (Table 1) that speed up hemoglobin oxidation from ferrous to ferric form.

**Table 1:** Drugs and substances that can result in the development of methemoglobinemia

Substance	Mechanism	Notes
Nitrites	Oxidize hemoglobin to methemoglobin	Found in certain medications (e.g., nitroglycerin), recreational drugs (e.g., poppers)
Nitroprusside	Releases cyanide ions, which oxidize hemoglobin	Used as a vasodilator in hypertensive emergencies
Aniline	Metabolized to toxic metabolites	Found in industrial chemicals and dyes
Dapsone	Metabolite (hydroxylamine) oxidizes hemoglobin	Used in the treatment of leprosy and dermatitis herpetiformis
Benzocaine	Oxidation of the heme iron	Commonly used as a topical anesthetic
Prilocaine	Oxidation of the heme iron	Local anesthetic agent
Lidocaine	Oxidation of the heme iron	Frequently used in dentistry and minor surgeries
Phenazopyridine	Oxidizes hemoglobin directly	Used to relieve urinary pain and discomfort
Sulfonamides	Metabolites (e.g., sulfanilamide) oxidize hemoglobin	Antibiotics with various medical uses
Naphthalene	Metabolite (naphthol) oxidizes hemoglobin	Found in mothballs and certain insecticides
Chlorates	Oxidize hemoglobin to methemoglobin	Found in certain herbicides and disinfectants
Metoclopramide	Metabolite (aminopropioaldehyde) oxidizes hemoglobin	Used to treat gastrointestinal disorders
Paraquat	Oxidative stress on red blood cells	Highly toxic herbicide
Nitrobenzene	Metabolite (nitrophenol) oxidizes hemoglobin	Used in the production of aniline and as a solvent
Amyl nitrite	Oxidize hemoglobin to methemoglobin	Used recreationally ("poppers") and medically (e.g., in cyanide poisoning)
Sodium nitrite	Oxidize hemoglobin to methemoglobin	Used in food preservation and as an antidote for cyanide poisoning
Isobutyl nitrite	Oxidize hemoglobin to methemoglobin	Used as a recreational drug and aphrodisiac
Glyceryl trinitrate	Oxidize hemoglobin to methemoglobin	Used to treat angina pectoris and heart failure
Phenytoin	Oxidize hemoglobin to methemoglobin	Anticonvulsant medication

Diagnosis of methemoglobinemia is typically confirmed through co-oximetry [2], which measures light absorption at four different wavelengths. Clinical suspicion can also be raised based on specific clinical indicators, such as refractory hypoxia, "cyanosis-saturation gap," and brown blood color [3]. While co-oximetry provides a definitive diagnosis, simple bedside tests can offer preliminary indications of methemoglobinemia.

Treatment of methemoglobinemia includes elimination of the causative agent, vitamin C, and methylene blue [4,5]. The use of methylene blue is indicated in symptomatic methemoglobinemia regardless of methemoglobin level and in cases where the methemoglobin level is above 30%. The drug is contraindicated in patients with glucose-6-phosphate dehydrogenase (G6PD) deficiency, and caution is required in patients at risk of developing serotonin syndrome. Methylene blue is a monoamine oxidase (MAO) inhibitor and can lead to the development of serotonin syndrome in interaction with other drugs.

Bone marrow biopsy findings in multiple myeloma (MM) are crucial for risk stratification and determining the Revised International Staging System (R-ISS) score. These findings include an assessment of plasma cell infiltration percentage, cytogenetic abnormalities (e.g., del(17p), t(4;14), t(14;16)), and other molecular markers. Risk stratification categorizes MM patients into standard, intermediate, and high-risk groups based on these findings, influencing treatment decisions and prognosis assessments.

## Conclusions

Methemoglobinemia can often be missed; if not diagnosed early, it can lead to confusion, seizures, coma, and even death. The diagnosis of methemoglobinemia relies on a clinical assessment grounded in the patient's history and presenting symptoms, particularly hypoxemia, which is unresponsive to supplemental oxygen. The term "refractory hypoxemia" serves as a key diagnostic indicator, often associated with a "saturation gap". There are only a few conditions causing the saturation gap (i.e., methemoglobinemia, CO poisoning, sulfhemoglobinemia, and peripheral vasoconstriction). In our patient's given history of recent dapsone use, methemoglobinemia was on top differential. Definitive diagnosis is established through arterial or venous blood gas analysis with co-oximetry, which enables the differentiation of hemoglobin types, determining the concentration of methemoglobin. It is crucial to note that percentage SpO_2_ measurements cannot be directly used to gauge the severity of methemoglobinemia. Timely identification of the severity and appropriate management are imperative to prevent complications. There is not a direct association between multiple myeloma and methemoglobinemia. However, certain medications used in the treatment of multiple myeloma, such as dapsone or some chemotherapy drugs such as cyclophosphamide and ifosfamide, can potentially cause methemoglobinemia as a side effect. Additionally, multiple myeloma patients may be at increased risk of infections or other medical conditions that could lead to methemoglobinemia. Diagnosing dapsone-induced methemoglobinemia in a patient with multiple myeloma presents several challenges due to overlapping symptoms with multiple myeloma complications such as confusion, cyanosis, and hypoxia, potential confounding medications, such as chemotherapy drugs and antibiotics, and the necessity of distinguishing methemoglobinemia from other causes of hypoxia. Laboratory tests to confirm methemoglobinemia can be complicated by concurrent anemia and other hematological abnormalities characteristic of multiple myeloma. Clinical judgment, thorough medication history review, close symptom monitoring, and interdisciplinary collaboration are essential for accurate diagnosis and appropriate management in these complex cases.
